# Hemorrhagic Complications Following Abdominal Paracentesis in Acute on Chronic Liver Failure: A Propensity Score Analysis

**DOI:** 10.1097/MD.0000000000002225

**Published:** 2015-12-11

**Authors:** Su Lin, Mingfang Wang, Yueyong Zhu, Jing Dong, Zhiyuan Weng, Lingyun Shao, Jing Chen, Jiaji Jiang

**Affiliations:** From the Liver Research Center (SL, MW, YZ, JD, JC, JJ); Cardiology Department of the First Affiliated Hospital of Fujian Medical University, Fuzhou (ZW); and Department of Infectious Diseases, Huashan Hospital of Fudan University, Shanghai, China (LS).

## Abstract

Patients with acute on chronic liver failure (ACLF) usually present with severe coagulopathy. Abdominal paracentesis is often performed in these patients. The aim of this study was to analyze the prevalence of hemorrhagic events after paracentesis and the predictive factors of this condition in ACLF populations.

ACLF patients who underwent paracentesis were retrospectively enrolled within a 5-year period. A propensity score (PS) matching analysis was used to select matched cases from the overall nonhemorrhagic group to be used as the control group. Hemorrhagic complications and risk factors were examined using logistic regression analysis.

A total of 602 abdominal paracenteses were carried out on 218 ACLF patients and 18 (2.99%) hemorrhagic complications were identified. The MELD scores were higher in hemorrhagic patients than overall patients before PS matching (25.77±6.65 vs 21.04 ± 7.93, *P* = 0.013). We matched 18 cases with bleeding events to 72 unique cases without. The hemorrhagic group had significantly lower fibrinogen levels and higher PT levels than nonhemorrhagic cases. Logistic regression analysis revealed that lower fibrinogen levels could independently predict hemorrhagic complications (OR: 0.128, 95% CI: 0.023–0.697, *P* = 0.017). The best cut-off value for reliable measurement of fibrinogen levels was 0.70 g/L, with a sensitivity of 76.4% and a specificity of 80.0%. The area under curve was 0.733 (95% CI 0.604–0.862, *P* value 0.002).

Severe hemorrhagic complications occur more commonly in ALCF patients than previously thought. A low fibrinogen level is an independent predictor of bleeding events in patients with MELD >25.

## INTRODUCTION

Abdominal paracentesis, either for diagnostic or therapeutic purposes, is frequently performed for patients with liver disease in the Gastroenterology or Emergency Departments. One severe complication of paracentesis is hemorrhage. Hemorrhage after paracentesis is occasionally documented in some case reports or case series studies. Populations with severe liver disease are frequently affected.^1,2^ The outcome of those patients with bleeding complication tends to be poor, with the 30-day mortality of 42.6%.^2^

Acute-on-chronic liver failure (ACLF) is a unique presentation of liver disease. It is defined as an acute deterioration of liver function in patients with chronic liver disease. ACLF patients display clinical characteristics of both chronic and acute liver dysfunctions, such as severe coagulopathy and ascites.^3^ The paracenteses are often performed in these patients. The traditionally held belief is that the bleeding risk of patients with liver failure exceeds that of general population or even patients with cirrhosis.^4^ Up to now, the complications of paracentesis for patients with acute liver failure or ACLF have not been well studied. Since the complication is rare, it is difficult to conduct prospective study or randomized trials addressing this issue. There is no data regarding the prevalence and risk factors of this severe complication.

To determine the answers to the questions posed above, we herein conducted a 5-year retrospective cohort study to analyze the incidence rate of severe bleeding complications following paracentesis and the predictive factors of this condition in patients with ACLF.

## METHODS

### Patients

In this retrospective longitudinal cohort study, the patients who were diagnosed with ACLF in the Liver Research Center of the First Affiliated Hospital of Fujian Medical University from 1 January 2010 to 31 December 2014 were enrolled. Patients who were pregnant or had spontaneous hemoperitoneum, hemoperitoneum secondary to blunt trauma, or any evidence of malignancy were excluded.

ACLF was diagnosed based on the consensus recommendations of the Asian Pacific Association for the study of the liver (APASL) as follows: acute hepatic insult manifested as jaundice and coagulopathy complicated within 4 weeks by ascites and/or encephalopathy in a patient with previously diagnosed or undiagnosed chronic liver disease.^5^ According to this definition, cirrhosis is not necessary for ACLF diagnoses.

Cirrhosis was diagnosed based on clinical features, including a history consistent with chronic liver disease as well as a documented complication of chronic liver disease (ie, ascites, varices, hepatic encephalopathy) and/or imaging consistent with cirrhosis.

The presence of ascitic fluid was confirmed on physical examination by the demonstration of shifting dullness or a fluid wave or by ultrasonography/radiology examination.

Organ damage was observed, especially for acute kidney injury (AKI). The diagnoses of AKI was based on the revised consensus the recommendations of the International Club of Ascites.^6^

### Data Collection

Coagulation parameters were routinely measured, and routine biochemical tests were conducted every 3 days for patients with ACLF. Clinical and historical data included the etiology of liver disease, co-morbidities, MELD score, Child–Pugh Score, and laboratory data (platelet count, PT, APTT, thrombin time (TT), and fibrinogen).

### Paracentesis

Patients who had any signs of ascites were subjected to routine paracentesis on admission. In case with signs or symptoms of peritonitis during hospitalization or a large volume of ascites that caused symptoms, the paracentesis was repeated.

Diagnostic paracentesis was performed with a 3.2-cm, 22-gauge needle to detect spontaneous bacterial peritonitis. Therapeutic paracentesis was performed when patients had large volumes of ascites to relieve abdominal tension or dyspnea. A 15-cm plastic catheter with multiple side holes and a 16-gauge metal introducer needle (Shanghai Shangri kangge Medical Instruments Co., Ltd.) or a 20-cm, 16-gauge intravenous plastic tubing with 18-gauge puncture needle (Guangdong Baihe Medical Technology Co., Ltd) was used for therapeutic paracentesis.

The patients were informed of the indications, risks, and alternatives before the procedures. Written informed consent for paracentesis was obtained from all patients. The procedures were performed by experienced residents or attending doctors in a dedicated paracentesis room or at bedside.

### Definition of Complications

A hemorrhagic complication was defined as a bleeding event in a patient who displayed no evidence of hemorrhagic ascites before the procedure and then began to demonstrate hemorrhagic ascites during or after paracentesis or abdominal hematoma within 7 days after the procedure^1,7–9^.

Intraperitoneal hemorrhage was defined as grossly bloody or homogeneously pink ascitic fluid with a red blood cell (RBC) count >10 × 10^9^/L that developed during or after paracentesis. The possibility of bacterial peritonitis or hemoperitoneal carcinomatosis was ruled out. Delayed intraperitoneal hemorrhage was defined as bloody ascites and was diagnosed by a repeated paracentesis. Acute hemorrhage was defined as grossly bloody ascites that appeared during paracentesis. Once the hemorrhagic complication was diagnosed, the subsequent paracenteses of that particular individual were not included in the analysis. According to this definition, 1 patient could only have 1 episode of bleeding complications in our study.

Abdominal wall hematoma was defined as a tender bruise surrounding the paracentesis site and was confirmed by ultrasound and/or computed tomography (CT) scan.

### Statistical Analysis

Each episode of paracentesis was considered as a separate event during statistical analysis. The number of hemorrhagic events was very low compared with the total paracentesis episodes. This difference might limit the robustness of the multivariable analysis with a high number of variables. Therefore we used propensity score (PS) matching analysis to select the matched cases.

Initially, we reviewed previous studies. It seemed that in cirrhotic patients, bleeding from direct puncture of a superficial abdominal wall vein and mesenteric varices 6 or the mesenteric variceal rupture after sudden release of abdominal wall pressure following paracentesis ^10,11^ might contribute to the hemorrhagic complications. Repeated puncture might increase the risk of vessel injury. In our previous study, hemorrhagic events seemed to happen in more severe cases.^12^ Therefore, we computed the propensity score using logistic regression with the independent variables including age, gender, presence of cirrhosis, MELD score, episodes of paracentesis, and volume of ascites removed. We matched each case of bleeding events to 4 nonbleeding cases with a propensity using greedy nearest neighbor matching. We were able to match 18 cases with bleeding events to 72 unique cases without bleeding complications.

Continuous variables were expressed as the mean with the standard deviation or the median with the interquartile range and categorical variables expressed as numbers (percentage) as appropriate. The Mann–Whitney test or Student *t* test was used for comparison between groups. The significance of difference in proportions was tested with the χ2-statistic. Factors that with *P* value <0.1 with hemorrhagic complications were entered into step-forward multivariate logistic regression and the odds ratio (OR) with 95% confidence intervals (95% CI) determined. The best cut-off values for risk factors were determined using receiver operating characteristic (ROC) curve analysis. Statistical significance was achieved if *P* < 0.05 with 2-tailed tests. All data were analyzed using the Statistical Package for Social Sciences (SPSS 18.0, Chicago, IL). The propensity score was computed by SPSS software with Integration Plug-In for R. The Kaplan–Meier curve was used to demonstrate the increasing risk of hemorrhagic events with the decline of fibrionogen levels. The missing data were excluded.

## ETHICS

The study protocol was approved by the Institutional Ethics Committee of the First Affiliated Hospital of Fujian Medical University and was in compliance with the Declaration of Helsinki.

## RESULTS

### Baseline Characteristics of Patients

**FIGURE 1:**
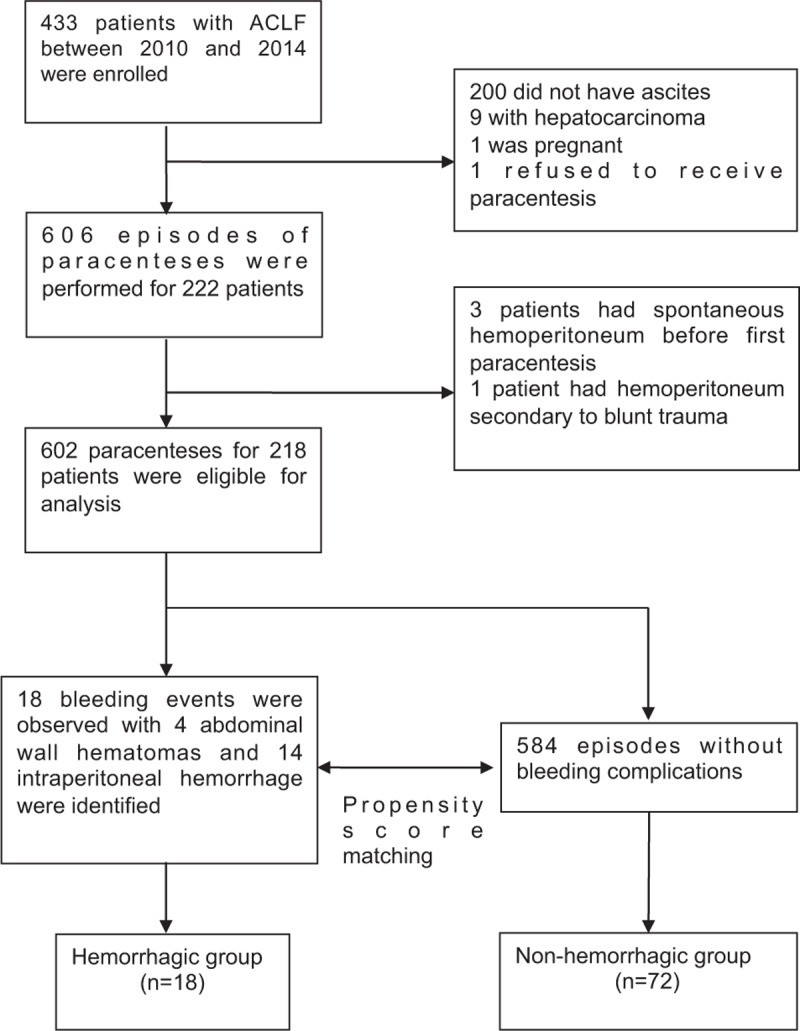
Flowchart of the study design.

During a 5-year period starting from January 2010, a total of 433 patients were diagnosed with ACLF. Three patients with spontaneous hemoperitoneum and 1 with hemoperitoneum secondary to blunt trauma were excluded. Two-hundred-eighteen patients who had ascites and received abdominal paracenteses were enrolled in this study (Fig. 1).

Among those 218 ALCF patients, 152 (69.7%) had underlying cirrhosis. There were 153 (70.2%) cases of hepatitis B virus (HBV), 21 (9.6%) cases of alcoholic hepatitis, 20 (9.2%) cases of combined HBV and alcoholic hepatitis, 5 (2.3%) cases of autoimmune liver disease, 4 (1.8%) cases of Wilson's disease, 2 (0.9%) cases of nonalcoholic fatty liver disease and 13 (6.0%) cases of liver cirrhosis of unknown cause.

A total of 602 abdominal paracenteses, including 371 (52.7%) diagnostic and 285 (47.3%) therapeutic, were carried out for these patients. The median episodes of paracenteses were 2 (1–12) with 50 (0–6900) mL of ascitic fluids removed. Forty episodes were dry with 0 mL removed. Eighty-eight patients died (40.4%) within 12 weeks of admission.

There were significant differences in the Meld scores, platelet counts, PT, INR, APTT, fibrinogen levels, and TT between the hemorrhagic group and the overall nonhemorrhagic groups. The MELD scores were higher in the hemorrhagic group than the overall group before PS matching (25.77 ± 6.65 vs 21.04 ± 7.93, *P* = 0.013). After PS matching, there were no significant differences in the MELD scores, platelet counts, INR, APTT, and TT between those 2 groups. The average MELD scores were 25.18 ± 7.64 in the nonhemorrhagic group and 25.77 ± 6.65 in the hemorrhagic group (*P* = 0.762). Table 1 shows the baseline characteristics of patients before and after propensity matching.

**TABLE 1:**
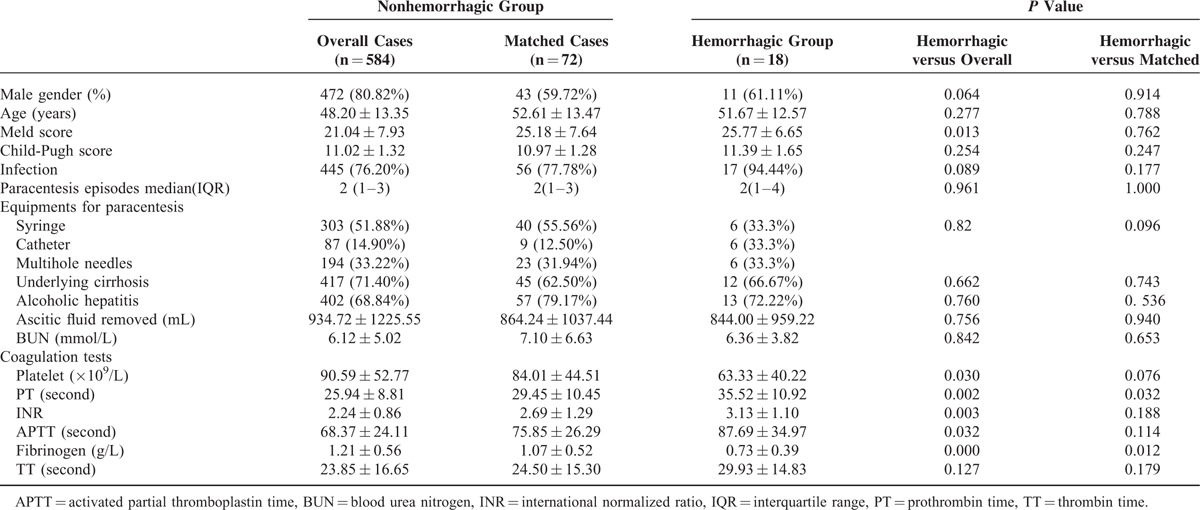
Baseline Characteristics of All Paracentesis Episodes Before and After Propensity Score Matching

### Clinical Course and Outcomes of Hemorrhagic Complications

A total of 18 hemorrhagic complications, including 4 abdominal wall hematomas and 14 intraperitoneal hemorrhages, were identified in 18 patients during 5 years. The details of those patients were shown in supplementary Table 1. Six of them received diagnostic paracentesis and the rest 12 patients were therapeutic ones. The puncture site was chosen at the one-third of the distance from the left anterior superior iliac spine to the umbilicus for all those 18 patients.

Hematomas were found within a median of 2 days (2–3) after paracenteses. Supplementary Figure 1 demonstrated a 74-year old female with abdominal wall hematoma after paracentesis. Eleven cases of delayed intraperitoneal hemorrhage were discovered 2 (1–3) days after paracentesis by a repeated paracentesis. Three cases of acute intraperitoneal hemorrhage took place within 30 min after the initiation of paracentesis. Most patients, especially those with hematomas, suffered from abdominal distension and pain. Six (33.3%) patients had symptoms of hypovolemia. Fifteen (83.3%) patients received plasma or platelet transfusions, and 9 (50.0%) required RBC transfusions. Acute kidney injury was found in 5 patients after hemorrhage, with 2 cases of stage 1and 3 cases of stage 2. A total of 13 (72.2%) in the hemorrhagic group died or received liver transplantation within 2 weeks after bleeding events. Mortality was related to the underlying liver disease and was not a direct result of hemorrhagic complications, except in 2 (11.1%) cases with uncontrollable intraperitoneal bleeding. Those 2 patients did not have any chance of receiving a liver transplant because of the economic burden and the shortage of donor organs. They refused to receive further treatment and eventually died.

### Risk Factors for Hemorrhagic Complications During Paracentesis in Patients With ACLF

The cases with hemorrhagic events had significantly lower fibrinogen levels and higher PT levels than did the nonhemorrhagic cases after PS matching (Table 1). The platelet count was lower in the hemorrhagic patients; however, the difference was not significant (*P* = 0.076).

**FIGURE 2:**
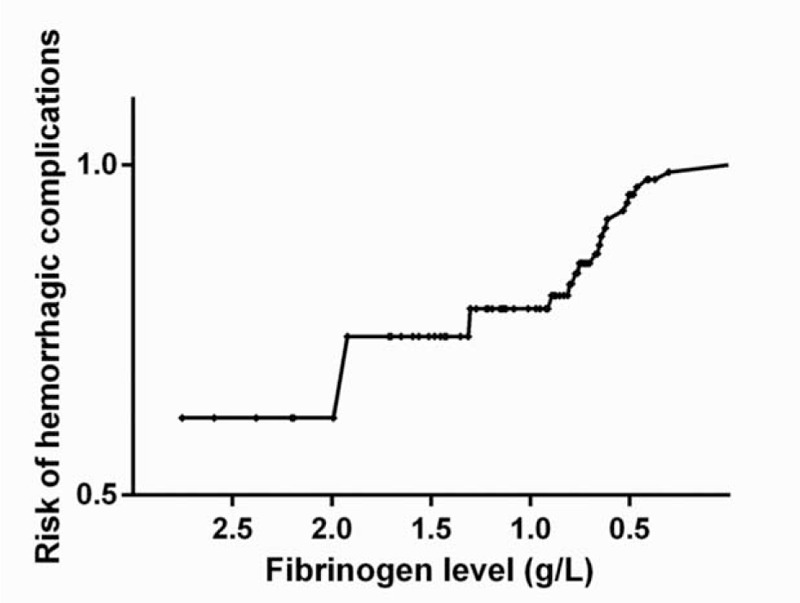
Kaplan–Meier analysis of the risk of hemorrhagic complications according to the fibrinogen levels.

Those variables with *P* values <0.1, including the PT, fibrinogen level, and platelet count, were selected for bilinear regression to determine the risk factors relevant to hemorrhagic complications. The result revealed that a lower fibrinogen level could independently predict hemorrhagic complication (OR: 0.128, 95% CI: 0.023–0.697, *P* = 0.017). The accumulated risk of hemorrhagic complications was increased with the decreasing fibrinogen levels (Fig. 2).

### The Predictive Value of Fibrinogen Levels for Hemorrhagic Complications

**FIGURE 3:**
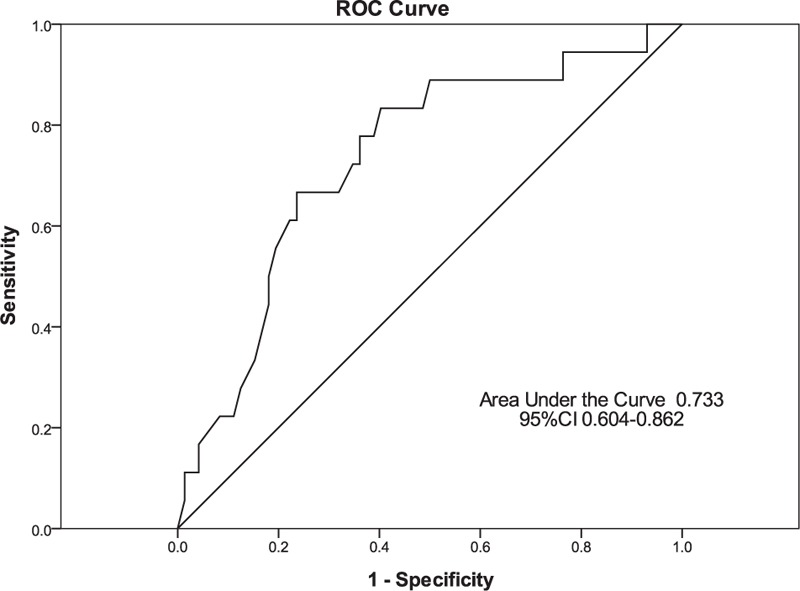
ROC curve of the fibrinogen levels for predicting hemorrhagic complications.

We used ROC analysis to evaluate the predictive value of fibrinogen. The area under the curve of fibrinogen for predicting hemorrhagic events was 0.733 (95% CI 0.604–0.862, *P* value 0.002). The best cut-off value was 0.70 g/L, with a sensitivity and specificity of 76.4% and 80.0%, respectively. The positive predictive value and the negative predictive value of fibrinogen were 36.5% and 91.9%, respectively (Fig. 3).

## DISCUSSION

This retrospective analysis of 602 consecutive abdominal paracentesis for patients with ACLF disclosed a much higher rate [2.99%(18/602)] of severe hemorrhagic complications compared with previous reports (under 1%).^1,7,13^ Patients with MELD scores > 25 were more likely to be affected. The bleeding events could result in kidney injury and poor outcomes.

Bleeding from direct puncture of a superficial abdominal wall vein or mesenteric varices in cirrhotic patients has been hypothesized to explain the hemorrhagic complications following abdominal paracentesis.^7^ In this study, the hemorrhagic and nonhemorrhagic groups had similar proportions of cirrhosis (62.50% vs 66.67%, *P* = 0.743), suggesting that variceal rupture might not play a major role. But interestingly, abdominal wall hematomas was only found in cirrhosis, which was in agreement with the results reported by Pache et al^1^ that only the patients with cirrhosis were attacked by hematomas. Directed injury of the dilated abdominal wall was possible to contribute to hemorrhagic events in cirrhotic patients. Paracenteses should be performed to patients with suspicious peritonitis as early as possible in order to rule out infection,^14^ detecting of portosystemic collateral veins by ultrasonographyor endoscopy could not be performed quickly. Tarantino *et al* suggested that the body mass index and blood ammonia may indicate the presence of collaterals.^15,16^ In these circumstances, these parameters might be helpful in differentiating patients with high hemorrhagic risk.

Patients with ACLF usually present characteristics of both chronic and acute liver impairment, which might lead to complex hemostatic dysfunction, with a prolongation of bleeding time, chronic coagulation activation, and hyperfibrinolysis.^3^ The platelet count is often decreased because of the precipitated cirrhosis and hypersplenism. The platelet count of the nonhemorrhagic cases was relatively higher than that of the hemorrhagic cases. However, after PS matching for the presence of cirrhosis, the difference between those 2 groups did not reach a significant level (84.01 ± 44.51 vs 63.33 ± 40.23, *P* = 0.076). Although the PT level was higher in hemorrhagic individuals than in nonhemorrhagic cases, it was not an independent risk factor for bleeding events after multivariate analysis. The results were consistent with previous studies in that they indicated that the PT and platelet count and routine hemostasis tests for coagulopathy failed to reflect the bleeding tendency in cirrhosis.^17–19^

In this study, the patient's fibrinogen level was the only independent predictor for hemorrhagic complications after paracentesis. Fibrinogen is synthesized in the liver by hepatocytes.^20^ Hypofibrinogenemia is observed in 40% patients with liver cirrhosis.^21^ Fibrinogen levels >1 g/L are sufficient to initiate coagulation and a low fibrinogen level often suggests hyperfibrinolysis.^18^ Some authors believed that hyperfibrinolysis might be the result of ascites.^21,22^ However, it is unclear whether fibrinogen levels are related to bleeding events in ACLF patients after paracentesis. In this ACLF patient cohort with MELD scores greater than 25, fibrinogen levels ≤0.70 g/L were strongly associated with hemorrhagic events after abdominal paracentesis. The positive predictive value was 36.5%, which means those populations might have a nearly 1/3 chance to suffer bleeding complications after paracenteses.

Although delayed paracentesis has been demonstrated to be associated with increased in-hospital mortality in patients with spontaneous bacterial peritonitis,^14^ but in our cohort, most bleeding events (12/18) occurred in patients with therapeutic ones. In a large retrospective cohort study of paracentesis on cirrhotic patients, major complications were associated with therapeutic procedures, not diagnostic ones.^19^ Terlipressin plus diuretic and albumin seemed effective in patients with refractory ascites in a multicentric study.^23^ Therefore we suggested that, instead of therapeutic paracentesis, less aggressive therapy as terlipressin could be used for patients with extremely low levels of fibrinogen to minimized bleeding complications.

There were some limitations of this study. At first, the retrospective nature might compromise the reliability of this finding. Second, the hemorrhagic complications could be under estimated because late intraperitoneal hemorrhage was diagnosed by a repeated paracentesis. Accordingly, asymptomatic patients with mild intraperitoneal hemorrhage might not be detected without a second paracentesis. It was worth noticing that 3 cases (in 18 cases) of bleeding occurring within 30 min of the paracentesis. It was hard to rule out the possibility of direct injury of the dilated abdominal wall or the mesenteric vein. Finally, this result only revealed a risk factor. We still did not know the exact reason for the bleeding events. Therefore, a well-designed prospective study should be conducted in the future to enrich this result.

## CONCLUSIONS

Severe hemorrhagic complications occur more commonly in ALCF patients than previously thought. A low fibrinogen level is an independent predictor of bleeding events in patients with MELD >25. Hence, the potential bleeding danger of this procedure should be emphasized for ACLF patients.
